# Updating the evidence: A systematic review of a decade of Infant and Early Childhood Mental Health Consultation (IECMHC) research

**DOI:** 10.1002/imhj.22033

**Published:** 2022-12-24

**Authors:** H. Callie Silver, Annie E. Davis Schoch, Alysse M. Loomis, Christen E. Park, Katherine M. Zinsser

**Affiliations:** ^1^ Center for Child and Human Development Georgetown University Washington D.C. USA; ^2^ College of Social Work University of Utah Salt Lake City Utah USA; ^3^ Department of Psychology University of Illinois Chicago Chicago Illinois USA

**Keywords:** consultation, early childhood care and education, infant and early childhood mental health, interventions, social‐emotional learning, cuidado y educación en la temprana niñez, aprendizaje socioemocional, intervenciones, consulta de salud mental, revisión sistemática, Soin et éducation de la petite enfance, apprentissage socio‐émotionnel, interventions, consultation de santé mentale, revue systématique, Frühkindliche Betreuung und Bildung, sozial‐emotionales Lernen, Interventionen, psychosoziale Beratung, systematisches Review, 乳幼児期のケアと教育, 社会的‐情動的学習, 介入, 精神保健相談, 系統的レビュー, 关键词:幼儿保育与教育, 社交情感学习, 干预, 心理健康咨询, 系统综述, **الكلمات المفتاحية**: رعاية وتعليم الطفولة المبكرة ، التعلم الاجتماعي العاطفي ، التدخلات ، استشارات الصحة النفسية ، المراجعة المنهجية

## Abstract

Infant and Early Childhood Mental Health Consultation (IECMHC) is a preventative, capacity‐building intervention in which mental health professionals partner with early childhood professionals to indirectly improve the environments and relationships that young children experience. Prior research has demonstrated that IECMHC is associated with positive outcomes for children, teachers, and classrooms. Over the past decade, IECMHC implementation and research have expanded, warranting an updated review. The current paper provides an update of the IECMHC evidence base. Included studies (*n* = 16) were systematically gathered, screened, and coded for context, intervention characteristics, methods and measures, outcomes across ecological levels, and alignment with the IDEAS Impact Framework's guiding questions. Our analysis replicates prior reviews, describing the positive impact of IECMHC on outcomes such as child externalizing behavior, teacher self‐efficacy, and teacher‐child interactions. Beyond updating prior reviews, this analysis describes emerging, nuanced findings regarding the mechanisms of change and the differential impact of IECMHC. We augment our review with descriptions of evaluations that did not meet our inclusion criteria (e.g., IECMHC in the home visiting context, unpublished evaluation reports) to provide context for our findings. Finally, we provide policy and practice implications and articulate an agenda for future research.

## Resumen

Introducción: La Consulta de Salud Mental Infantil y la Temprana Niñez (IECMHC) es una intervención preventiva de capacidad máxima en la que profesionales de la salud mental y los de la temprana niñez trabajan juntos para mejorar indirectamente los ambientes y relaciones que experimentan los pequeños niños. Revisiones anteriores reportaron que IECMHC se asocia con resultados positivos para niños, maestros y la sala de clases (Brennan et al., 2008; Perry at al., 2010). A lo largo de la pasada década, la implementación e investigación de IECMHC se ha expandido, lo cual garantiza una revisión actualizada.

Métodos: El presente ensayo aporta una actualización de IECMHC con base en evidencia. Los estudios incluidos (n = 16), de manera sistemática se recogieron, se evaluaron y se codificaron por contexto, características de intervención, métodos y medidas, resultados a lo largo de niveles ecológicos y alineamiento con las preguntas guías del Marco de Impacto IDEAS.

Resultados: Nuestro análisis reproduce exactamente revisiones anteriores, describiendo el positivo impacto de IECMHC sobre resultados tales como la conducta de externalización del niño, la auto efectividad del maestro y las interacciones maestro‐niño. Además de actualizar las revisiones previas, este análisis describe los salientes y matizados resultados con respecto a los mecanismos de cambio y el impacto diferencial de IECMHC.

Conclusión: Aumentamos nuestra revisión con descripciones de evaluaciones que no reunieron los criterios para ser incluidas (v.g., IECMHC dentro del contexto de visitas a casa, reportes de evaluación no publicados) con el fin de aportar un contexto a nuestras observaciones. Finalmente, aportamos implicaciones para las políticas y la práctica y presentamos una agenda para la futura investigación.

## Résumé

Introduction: La Consultation de la Santé Mentale du Nourrisson et de la Petite Enfance (en anglais Infant and Early Childhood Mental Health Consultation, soit IECMHC) est une intervention de prévention et de renforcement des capacités grâce à laquelle les professionnels de la santé mentale forment un partenariat avec les professionnels de la petite enfance pour indirectement améliorer les environnements et les relations dont les jeunes enfants font l'expérience. Des revues précédentes ont montré que l'IECMHC est liée à des résultats positifs pour les enfants, les enseignants et les salles de classe (Brennan et al., 2008; Perry et al., 2010). Au fil des dix dernières années la mise en place de l'IECMHC et les recherches se sont étendues, exigeant une actualisation de la revue.

Méthodes: Cet article offre une mise à jour des preuves de l'IECMHC. Les études inclues (n = 16) ont été systématiquement recueillies, examinées et codées pour ce qui concerne le contexte, les caractéristiques de l'intervention, les méthodes et les mesures, les résultats au travers des milieux écologiques, et l'alignement avec les questions directrices de la structure IDEAS Impact Framework.

Résultats: Notre analyse réplique les revues précédentes, décrivant l'impact positif de l'IECMHC sur les résultats tels que le comportement d'externalisation de l'enfant, l'auto‐efficacité de l'enseignant, les interactions enseignant‐enfant. Au delà de la réactualisation de revues précédentes cette analyse décrit des résultats émergeants et nuancés pour ce qui concerne les mécanisme de changement et l'impact différentiel de l'IECMHC.

Conclusion: Nous développons notre revue avec des descriptions d’évaluations qui n'avaient pas rempli les critères d'inclusion (par exemple l'IECMHC dans le contexte de la visite à domicile, des rapports d’évaluation non publiés) afin d'offrir un contexte à nos résultats. Enfin, nous offrons des perspectives sur les implications pour les décisions et la pratique et articulons un agenda de recherches futures.

## Zusammenfassung

Aktualisierung der Evidenz: Ein systematisches Review über ein Jahrzehnt der Forschung zur Beratung zur psychischen Gesundheit von Säuglingen und Kleinkindern (Infant and Early Childhood Mental Health Consultation; IECMHC)

Einleitung: Die Beratung zur psychischen Gesundheit von Kindern und Kleinkindern (Infant and Early Childhood Mental Health Consultation; IECMHC) ist eine präventive, kapazitätsbildende Intervention, bei der Fachkräfte aus dem Bereich der psychischen Gesundheit mit Fachkräften aus dem Bereich der frühen Kindheit zusammenarbeiten, um indirekt das Umfeld und die Beziehungen von Kleinkindern zu verbessern. Frühere Studien haben gezeigt, dass IECMHC mit positiven Outcomes für Kinder, Lehrkräfte und in Klassenräumen assoziiert ist (Brennan et al., 2008; Perry et al., 2010). In den letzten zehn Jahren hat sich die IECMHC‐Implementierung und ‐Forschung ausgeweitet, so dass ein aktualisiertes Review gerechtfertigt ist.

Methoden: Die vorliegende Arbeit bietet eine Aktualisierung der IECMHC‐Evidenzbasis. Eingeschlossene Studien (n = 16) wurden systematisch gesammelt, gesichtet und hinsichtlich des Kontextes, der Interventionscharakteristika, der Methoden und Maßnahmen, der Outcomes auf verschiedenen ökologischen Ebenen und der Übereinstimmung mit den Leitfragen des „IDEAS Impact Framework“ kodiert.

Ergebnisse: Unsere Analyse repliziert die Ergebnisse früherer Studien und beschreibt die positive Wirkung von IECMHC auf Outcomes wie externalisierendes Verhalten von Kindern, Selbstwirksamkeit von Lehrkräften und Lehrkraft‐Kind‐Interaktionen. Über die Aktualisierung früherer Reviews hinaus beschreibt diese Analyse neue, differenzierte Erkenntnisse über die Mechanismen der Veränderung und die unterschiedlichen Auswirkungen der IECMHC.

Schlussfolgerung: Wir ergänzen unser Review mit Beschreibungen von Evaluationen, die unsere Einschlusskriterien nicht erfüllten (z.B. IECMHC im Kontext von Hausbesuchen, unveröffentlichte Evaluationsberichte), um Kontext für unsere Ergebnisse zu liefern. Abschließend werden Implikationen für Politik und Praxis aufgezeigt sowie eine Agenda für zukünftige Forschung formuliert.

## 抄録

エビデンスの最新化

乳幼児期の精神保健相談 (IECMHC) 研究の 10 年間の系統的レビュー

はじめに 乳幼児期のメンタルヘルス相談 (IECMHC) は、予防的で能力構築的な介入であり、乳幼児が経験する環境と関係性を間接的に改善することを目的として、メンタルヘルス専門家が乳幼児の専門家と密に連携して行われるものである。先行研究は、IECMHCは子ども、教師、教室のポジティブな結果と関連していると報告してきた(Brennan et al., 2008; Perry et al., 2010)。過去10年間で、IECMHCの実施と研究は拡大し、最新のレビューが必要とされるようになった。

方法 本論文は、IECMHCの最新のエビデンスを提供する。含まれる研究 (n = 16) は、文脈、介入の特徴、方法と尺度、生態学的レベルにわたる成果、およびIDEASインパクトフレームワークの指針にそった質問との整合性について、系統的に収集、選別、コード化された。

結果 我々の分析は、先行研究を再現し、IECMHCは子どもの外在化行動、教師の自己効力感、教師‐子どもの相互交流といった成果に対して肯定的な影響があると記述している。先行研究を最新化するだけでなく、本分析では、変化のメカニズムやIECMHCの影響の違いについて、新たな微妙な知見を記述している。

結論 我々は、我々のレビューに、私達の知見に文脈を作るための包含基準をみたさない評価 (例えば、家庭訪問の文脈におけるIECMHC、未発表の評価レポート) に関する記述を加えた。最後に、政策と実践への示唆を与え、今後の研究の議題を明確にする。

## 摘要

研究背景:“婴幼儿心理健康咨询” (IECMHC) 一种预防性的能力养成干预措施, 也即心理健康专业人员与幼儿专业人员合作, 间接改善幼儿体验的环境和关系。之前的综述报告称, IECMHC与儿童、教师和教室的积极成果相关 (Brennan et al., 2008; Perry et al., 2010) 。在过去的十年中, IECMHC的实施和研究已经扩大化, 需要进行更新的综述。

研究方法:本论文是对IECMHC证据库的更新。对纳入的研究 (n = 16) 进行了系统收集、筛选和编码, 包括背景、干预特征、方法和措施、跨生态层面的结果以及与“IDEAS影响力框架”指导问题的一致性。

研究结果:我们的分析重复了之前的综述, 描述了IECMHC对儿童外化行为、教师自我效能和师生互动等结果的积极影响。除了更新之前的综述, 本分析还描述了有关IECMHC变化机制和差异影响的新的、细微的发现。

研究结论:我们通过对不符合纳入标准的评估 (例如, 家访环境中的IECMHC、未发表的评估报告) 的描述来补充我们的综述, 为我们的研究结果提供背景。最后, 我们提出了政策和实践启示, 并阐明了未来研究的议程。

## ملخص

مقدمة: استشارات الصحة النفسية للرضع والطفولة المبكرة(IECMHC) هي تدخل وقائي لبناء القدرات يشترك فيه أخصائيو الصحة النفسية مع متخصصي الطفولة المبكرة لتحسين البيئات والعلاقات التي يتعامل معها الأطفال الصغار بشكل غير مباشر. أفادت المراجعات السابقة أنIECMHC مرتبط بنتائج إيجابية للأطفال والمعلمين والفصول الدراسية(برينان وآخرون 2008 – بيري وآخرون 2010). و على مدى العقد الماضي ، توسع تطبيق (IECMHC) وأبحاثه ، مما يستدعي مراجعة محدثة. طريقة البحث: تقدم الورقة الحالية تحديثًا لقاعدة بيانات أدلة الاستشارات الصحة النفسية للرضع والأطفال. تم جمع الدراسات المضمنة (ن = 16) بشكل منهجي وفحصها وترميزها من حيث السياق وخصائص التدخل والأساليب والقياسات والنتائج عبر المستويات البيئية والمواءمة مع الأسئلة التوجيهية لإطار العمل. النتائج: يكرر تحليلنا المراجعات السابقة ، ويصف التأثير الإيجابي لـIECMHC على النتائج مثل سلوك الطفل الخارجي ، والكفاءة الذاتية للمعلم ، والتفاعلات بين المعلم والطفل. إلى جانب تحديث المراجعات السابقة ، يصف هذا التحليل النتائج الناشئة الدقيقة فيما يتعلق بآليات التغيير والتأثير المتفاوت لـ IECMHC. الاستنتاج: تم تعزيز المراجعة بأوصاف التقييمات التي لم تفي بمعايير التضمين وذلك لتوفير سياق لنتائج المراجعة. أخيرًا ، نقدم تطبيقات السياسات والممارسات ونضع أجندة للأبحاث المستقبلية.

## 1 INTRODUCTION

Infancy and early childhood are times of unparalleled risk and opportunity in an individual's developmental trajectory (Shonkoff, 2012), so interventions that buffer young children against negative influences and promote their strengths may have outsized impacts in the long term (Heckman, 2006). A range of programs has been developed to promote infant and early childhood mental health (IECMH), which refers to children's early social and emotional development, including relationship formation, self‐regulation, and learning/exploration (Johnston & Brinamen, 2006). IECMH is firmly grounded in trusting relationships with adults (e.g., parents and caregivers).

One such intervention, Infant and Early Childhood Mental Health Consultation (IECMHC), has expanded rapidly and now is prevalent in all 50 states. This relational approach is touted by federal and state advocates and policy‐makers for its role in improving the quality of early childhood programs and for being one of the only known ways to prevent early childhood expulsion and suspension (U.S. Department of Education & U.S. Department of Health & Human Services, 2014; U.S. Department of Health and Human Services, 2016). As the intervention has grown, so has the literature base, and yet, no comprehensive review of the evidence or its effectiveness has been conducted in the past decade. The present review aims to systematically review and synthesize the evidence of IECMHC published since the last reviews (Brennan et al., 2008; Perry et al., 2010). Further, we go beyond a descriptive presentation of findings to evaluate whether and how the literature adheres to recent advances in conceptualizations of program evaluation.

### 1.1 Infant and Early Childhood Mental Health Consultation

IECMHC is an indirect, preventative, capacity‐building intervention in which mental health professionals, with strong foundations in infant and early childhood mental health, partner with early childhood practitioners to enhance their skills in fostering social and emotional competencies in young children. IECMHC can be implemented in any setting in which young children under six years old grow and learn, including early childhood care and education (ECCE) settings, home visiting programs, primary care, and child welfare.

**KEY FINDINGS**

- A growing body of literature suggests that IECMHC has a positive impact in early childhood care and education settings across various ecological levels.
- The field has begun to explore mediators and moderators of IECMHC's success.
- There is a critical need for more disaggregated data and attention to demographic variability to better understand IECMHC's impact on young children who are at the greatest risk of suspension and expulsion.

**STATEMENT OF RELEVANCE**

Infant and Early Childhood Mental Health Consultation implementation and research has expanded widely and rapidly over the past decade. As more states look to this intervention to curtail pressing issues within the field of early childhood, it is critical that this evidence base be accessible and current. This review also serves to inform the research community as to necessary future directions to better understand nuanced elements of this intervention to maximize positive outcomes across diverse populations.

The consultant partners with the professional (e.g., educator, home visitor, early interventionist) to create a reflective space in which the adult can learn relevant skills, process challenges, and improve their interactions with children (Cohen & Kaufman, 2005). The activities of IECMH consultants can take many different forms but tend to include meeting with early childhood professionals and leaders to discuss challenging child behaviors, directly observing children's behaviors, working alongside professionals implementing new social‐emotional support strategies, and collaborating with families (Cohen & Kaufman, 2005). By embodying the “consultative stance,” the IECMH consultant remains nonjudgmental and curious about all levels of influence on both the child and adult (Johnston & Brinamen, 2006), understanding that children's behavior can be symptomatic of their environment (Division for Early Childhood of the Council for Exceptional Children, 2017).

The emphasis on multiple, reciprocal levels of influence on children is aligned with Urie Bronfenbrenner's Bioecological Systems theory (Bronfenbrenner, 1979, 1994). Just as this theory contextualizes a child's presentation within a network of environmental factors – including family, school, society, and culture – IECMHC is a multilevel intervention that addresses multiple layers of environmental influences. IECMH consultants who are asked to assist with a child's challenging behaviors often intervene in more systemic issues, such as communication challenges between parents and teachers, leadership issues that affect staff well‐being, crowded classroom spaces, and high rates of teacher turnover.

Because IECMHC targets multiple intersecting levels of influence, the outcomes of IECMHC must be measured across these levels. The most common setting for IECMHC is ECCE, and most studies of IECMHC are conducted in this setting. ECCE settings are rich ecologies and IECMHC can act on and have effects at its multiple levels including: child, family, teacher, classroom, program, and director. The current review mirrors the approach of the intervention itself by synthesizing outcomes at each of these various ecological levels.

### 1.2 The evidence base for Infant and Early Childhood Mental Health Consultation

More than a decade ago, two systematic reviews of the evidence base for ECCE‐based IECMHC summarized the landscape of IECMHC evidence. Perry et al.’s (2010) synthesis described the evidence for child‐level outcomes, while Brennan et al.’s (2008) synthesis described teacher‐, classroom‐, and program‐level outcomes. In their review, Perry and colleagues gathered all peer‐reviewed IECMHC outcomes studies from 1985 to 2008 with a randomized‐control trial design and/or a comparison group. In the 14 studies that met those inclusion criteria, all were ECCE‐based, although consultation was implemented with some variability across sites (e.g., length of consultation, consultant qualifications). Results from the qualitative content analysis revealed that IECMHC was consistently associated with reductions in teacher‐rated child externalizing behaviors (12 out of 13 studies that measured this construct) and improvements in teacher‐rated prosocial capacities (8 out of 9 studies). Change in children's internalizing behaviors was assessed less often, and findings were mixed (improvements reported in 3 out of 6 studies).

Brennan and colleagues’ review included 26 published and unpublished IECMHC outcomes studies (pre‐experimental, quasi‐experimental and randomized‐controlled trials) from 1985 to 2008 that addressed the staff‐ and program‐level outcomes affected by consultation. Regarding teacher outcomes, Brennan and colleagues reported that IECMHC was associated with increased staff self‐efficacy (9 out of 11 studies that measured this construct), competence in managing challenging behaviors of young children (8 out of 10 studies), improved staff sensitivity in interactions with children (4 out of 5 studies), and decreased job‐related stress (3 out of 4 studies). In terms of programmatic outcomes, IECMHC was associated with reduced staff turnover (5 out of 6 studies), and some studies (5 out of 8 studies) reported increased quality of childcare setting, typically measured with the Early Childhood Environment Rating Scale, Revised (ECERS‐R; Harms et al., 1998).

Together, these two reviews provided a solid foundation of evidence supporting the effectiveness of IECMHC across multiple levels. Nevertheless, additional synthesis of the IECMHC evidence is warranted for various reasons. First, there has been significant growth in the IECMHC evidence base in the past decade, so these syntheses are no longer current. This need was partially met by Albritton and colleagues’ (2019) systematic review, in which they analyzed the evidence for the impact of consultation on disproportionate rates of preschool discipline. Though this study made a meaningful and necessary contribution to the field, it focused on just one outcome (disproportionality in child exclusionary discipline) at one level (the child level) of consultation, and it included a broader array of consultation interventions, some of which did not meet Cohen & Kaufmann's original definition of IECMHC (2005). Hence, there remains a need for a review that captures a wider range of outcomes and adheres to a stricter definition of IECMHC.

In addition to updated information on IECMHC outcomes, more nuanced, data‐driven insights into IECMHC are needed. The authors of both reviews (Brennan et al., 2008; Perry et al., 2010) provided suggestions for future research, some of which have been undertaken in the intervening years, so an updated review is needed to summarize these findings. Specifically, the authors called for studies of: the role of cultural factors in moderating IECMHC outcomes; the constructs that mediate IECMHC outcomes in the theory of change; the key elements of IECMHC; and the possible causal link between short‐term and/or programmatic changes and longer‐term and/or child‐level changes.

To determine the extent to which recent studies of IECMHC address these calls for action, the present review will additionally examine their fidelity to modern, nuanced conceptualizations of program evaluation. For this study, we are leveraging the IDEAS Impact Framework to operationalize this adherence (Center on the Developing Child et al., 2016).

### 1.3 IDEAS Impact Framework

Motivated by a joint imperative to address the fact that not all children benefit equally from interventions, the IDEAS Impact Framework was developed through a collaboration among the Center on the Developing Child at Harvard University, the University of Oregon Center for Translational Neuroscience, and the University of Washington College of Education. These experts developed the IDEAS Impact Framework to help teams use data to understand and improve the impact of their interventions so that more children achieve better outcomes. The Framework is guided by the following questions: *What* about it works?*; How* does it work?; *For whom* does it work, and for whom does it *not work?*; and *In what contexts* does it work? (Center on the Developing Child et al., 2016).

More specifically, for interventions to move from modest to more robust impacts on children and families, this framework contends that they must investigate not only whole‐group changes from baseline to post‐intervention, but they also must investigate more granular, disaggregated findings. Statistically, the framework also encourages looking beyond main effects to parsing the moderators and mediators of the impact of an intervention. Such analyses are critical for assessing the impact of an intervention on existing disparities because analyses can assess whether an intervention is equally effective for all children or whether it has a differential impact on children based on variables known to reveal disparities, including race/ethnicity and socioeconomic status.

### 1.4 The present study

The present study builds upon prior reviews by systematically gathering IECMHC research published in the last decade and applying a modern framework in which to evaluate the evidence base. Systematic reviews are a powerful resource and can both guide future investment and highlight gaps requiring further inquiry. With the current review, we seek to not only describe what current research tells us about the continuous development of these interventions, but also to identify areas where greater evidence and specificity are needed. Beyond replicating the descriptive approaches used in the prior reviews, our study incorporates the IDEAS Impact Framework as an organizing structure to illustrate new insights and remaining gaps uncovered by IECMHC research.

Ultimately, this review not only describes the evidence base for multilevel outcomes associated with IECMHC, but seeks to shed light on the drivers of change and the potential mechanisms for the differential impact of consultation programs. Specifically, we seek to address the following research aims: 1) Describe the characteristics of IECMHC interventions with published evaluations between 2009–2020 and the contexts in which they are being implemented; 2) Describe the methods and measures employed in the evaluation of IECMHC interventions; 3) Synthesize the evidence regarding the main effects of IECMHC across ecological levels; and 4) Investigate the extent to which these modern evaluations align with the questions posed in the IDEAS Impact Framework.

## 2 METHODOLOGY

### 2.1 Literature search

**FIGURE 1:**
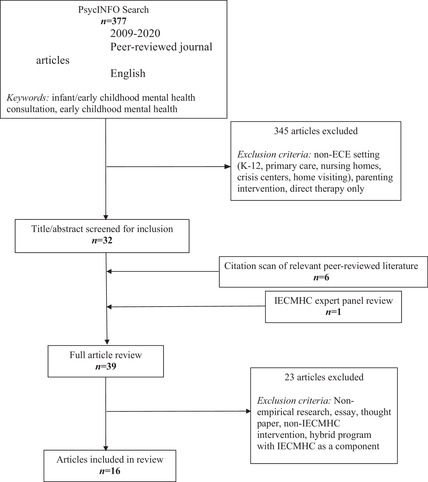
CONSORT diagram of literature search (2009 –December 2020).

We devised a search methodology and inclusion criteria based on those employed in prior reviews on this topic (Brennan et al., 2008; Perry et al., 2010). The procedures detailed below are summarized in the CONSORT flow diagram in Figure 1. Search terms included: infant/early childhood mental health consultation OR early childhood mental health consultation OR infant mental health consultation. Our search parameters included: peer‐reviewed journal, written in English, and conducted between 2009 (to minimize overlap with the previous systematic literature review) and December 2020. We conducted parallel searches in two electronic databases (PsycINFO and Web of Science). Although both databases yielded results, the Web of Science search yielded no unique articles and was fully redundant with the PsycInfo results that were retained.

### 2.2 Abstract screening

In total, our searches yielded 377 articles published between 2009 and 2020. These were screened by three members of the research team for adherence to our inclusion criteria. To be included in this review, papers needed to (a) be peer‐reviewed journal articles published in English between 2009 and December 2020; (b) report original, empirical research related to outcomes at any ecological level; and (c) report on data from mental health consultation in U.S.‐based settings serving children ages birth through six. Importantly, we used Cohen and Kaufmann's (2005) broad definition of consultation and therefore excluded studies reporting solely on direct therapy to children but included reports where consultants provided direct child‐specific services, so long as they were not the sole services provided. Finally, to focus specifically on the impacts of consultation, abstracts were excluded if IECMHC was only one component of a larger set of interventions being implemented and jointly evaluated. For example, in two studies we found, (Downer et al., 2018; Raver et al., 2009), manualized interventions were evaluated, of which IECMHC was only one element, and its effects could not be isolated. However, studies were screened in if consultation was added in addition to existing programming, curricula, or policies in a center (e.g., universal social‐emotional learning curricula).

Using these criteria, the majority of abstracts were excluded (*n* = 345) because the research was conducted outside of early childhood settings (e.g., K‐12 schools, nursing homes, crisis centers). Before advancing to the full‐text screening stage, we scanned the reference lists of the remaining 33 articles to identify any other relevant articles not originally captured in the online database searches. This process yielded six new articles to screen (Emde, 2009; Ocasio et al., 2015; Raver et al., 2011, 2009; Vinh et al., 2016; Ysseldyke et al., 2012). The list of abstracts meeting our screening criteria was then reviewed by the second and third authors, both experts in the field of IECMHC. They identified one additional paper missing from the list (Bender et al., 2017) that was published in the *Journal of Education and Developmental Psychology*. Although the study uses a U.S.‐based sample and meets all other requirements, this paper did not appear in the PsycINFO search results because the journal is funded by the Canadian Center for Science and Education. A targeted search of this journal using the same search terms listed above yielded no additional relevant papers meeting our criteria. We opted to further screen this article even though it was not identified by our search criteria, to ensure that our review was as up‐to‐date and comprehensive as possible.

### 2.3 Full‐text screening and coding

Two authors completed full‐text reviews to screen, and then subsequently coded the analysis set. Articles were screened for inclusion using the same inclusion criteria listed at the abstract stage. The coders each screened the articles separately and then met to discuss disagreements and finalize a consensus of articles to be included. These discussions also led to the refinement of the inclusion criteria, for example, the decision to not include articles that investigated interventions where the effects of IECMHC could not be isolated. Forty articles were reviewed fully and of these, 23 were excluded for one or more of the following reasons: did not include empirical, quantitative research findings (*n =* 13); did not report on IECMHC‐related outcomes (*n =* 12); evaluated multifaceted interventions so the effects of IECMHC could not be isolated (*n =* 6); IECMHC occurred in a non‐ECCE setting (*n =* 2); or intervention involved only direct therapy to children (*n =* 3).

### 2.4 Coding procedures and definitions

Articles in the final analysis set (*n =* 16) were then coded using procedures similar to those of the previous reviews (Brennan et al., 2008; Perry et al., 2010). For each article, two coders were responsible for completing a full‐text review to identify several study elements. When discrepancies between coders arose, the full team of authors met to discuss and come to an agreement. Specifically, coders contributed to content matrices summarizing various elements of the included articles. In Table 1, we report on the IECMHC Intervention Description/Setting (name of intervention, if applicable, type of ECCE program(s)), Intervention Activities (depending on what was provided in the article, this includes specific activities and/or their conceptualization of IECMHC), and Duration/Dosage (in the way it was originally presented by study authors). Table 2 includes information on the Sample (number of teachers, consultants, children, families, etc., when applicable), Study Design/Analysis (specifics on the type of study and analyses used), Outcomes Measured (metrics used and who the information was in parentheses), and Findings (both significant and non‐significant, when reported).

**TABLE 1:** Characteristics of evaluated IECMHC interventions

Study	Intervention description/setting	Intervention activities	Duration/ Dosage
Allen et al. (2012)	The present study was a secondary data analysis of a national survey of rural and urban Head Start staff and mental health consultants. The study uses a subset of the data used in Green et al. (2006)	Though mental health consultation is mandated in Head Start, the exact nature of services and activities differed by site. The authors conceptualize IECMHC in terms of child‐level and program‐level activities	n/a
Beardslee et al. (2010)	The Family Connections intervention utilized mental health consultation to help early childhood program staff to address mental health needs of the children and families they work with, with a specific focus on parental depression	Activities included direct services for parents (workshops, groups), working with groups of children, collaboratively‐planned staff trainings, and classroom observations to work with teachers and children	3 years (2x per week)
Bender et al. (2017)	The Child Care Expulsion Program (CCEP) is a state‐funded IECMHC program that provides child and family centered and programmatic consultation to programs in the Midwest to prevent expulsion of young children and promote social‐emotional development	Consultation with centers and programs (activities not specified)	3–6 months, ∼1‐3 h per week
Conners‐Burrow et al. (2012); Virmani et al. (2013)	This study was based on a three‐year IECMHC pilot project in Arkansas beginning in 2004. Three community mental health centers were funded to partner with early education programs in their region	Consultant activities differed by site but the majority were focused on teachers (and parents), as opposed to directly targeting the child. Mental health consultant activities were standard and included trainings, observations, meetings with teachers and families, and behavioral plans. Consultants also helped teachers design and conduct small and large group activities aimed to strengthen social skills	3 years of fall to spring (1 half day per week)
Conners‐Burrow et al. (2013)	See Conners‐Burrow et al. (2012) for model, though the setting of the intervention changed from Head Start and state‐funded Pre‐K programs to private childcare centers	See Conners‐Burrow et al. (2012)	6–8 months (weekly to monthly activities)
Conners‐Edge et al. (2020)	IECMHC was delivered to a subset of ECCE programs involved in a statewide expulsion prevention system. 28.4% of total cases in the system received IECMHC. Participating programs were all center‐based, but included child care, state funded pre‐K, and Head Start/Early Head Start	Consultation was provided by 10 master's level mental health professionals who received IECMHC‐specific training, ongoing reflection, and continuing education.Specific activities of the IECMHC were not provided	3 months
Crusto et al. (2013)	The goal of the Classroom Consultation for Early Childhood Educators Program (CCP) in Bridgeport, CT was to help teachers understand emotional development and behaviors that children exhibit and to support children's social‐emotional development	A triaged approach was used to provide universal classroom and child‐specific strategies, short‐term parent support and education, and home‐based intensive intervention	∼6 months
Davis et al. (2020)	The current program is a 4‐year statewide implementation of IECMHC in center‐based child care centers in a southwestern state	Program activities included: individual consultation meetings and observations of individual children	1x/ week
Gilliam et al. (2016)	Connecticut's statewide Early Childhood Consultation Partnership (ECCP) is a state‐funded program available to staff at all public and private early care and education centers serving children ages birth through age five in CT. Centers were mostly community‐based childcare, with a small portion of Head Start and public schools	This model focuses on the overall social‐emotional classroom environment, behavioral plans for individual children (involving parents), and classroom‐wide behavior management. Activities are manual‐based, but menu‐driven based on the individual needs of teachers and classrooms	2 months 4–6 h/week
Green et al. (2012)	Through an individually‐tailored program‐level intervention, mental health consultants sought to enhance 2 Head Start centers’ organizational capacity to support children's social‐emotional development	Program activities included: improving IECMHC services, mental health specific strategic planning, training to all staff in IECMHC best practices, and engaging programs in wellness activities to reduce staff stress and promote a healthy center climate	2 years
Heller et al. (2011); Heller et al. (2012)	An IECMHC program implemented in Louisiana for all children in center‐based care, not just those exhibiting behavior problems	The model included classroom observations, in‐class modeling, meetings with teachers, didactic group meetings, meetings with families, planning of specific child interventions, parent education, and referrals to outside services	6 months (6 hours per week, average 72 h total)
Silver & Zinsser, 2020	Survey of IECMHC use in community‐based preschools in a large Midwestern city	Teachers were asked if they have “received consultation from a mental health professional” however no specific program activities were defined for teachers	Past year
Upshur et al. (2009)	Together for Kids was a pilot demonstration project that blended program‐focused and individual child‐ and family‐focused services	Program‐focused activities included: observing classrooms and providing in‐classroom modeling of how to address children's behavior, delivering training sessions, and assisting programs in creating center‐wide parent education activities. Direct therapeutic services for high risk children and families were: individualized services (e.g. observations, therapy, meetings)	4–6 months (24.5 h average per case)
Vuyk et al. (2016)	This study aimed to evaluate a consultation intervention in a rural, applied setting in Southwestern Kansas. The model was based on the Georgetown IECMHC model, beginning with the consultant and director or provider clarifying the purpose of services and introducing the consultant to staff	Consultants conducted traditional IECMHC activities at the programmatic level primarily, with child‐level services available as needed. In participating preschool classrooms, two evidence‐based programs were used to foster children's social‐emotional skills	∼5 months (weekly or bi‐weekly meetings, ranging from 1–16 sessions)

**TABLE 2:** Methods used in IECMHC evaluation studies

Study	Sample	Study design/analysis	Outcomes measured (informant)	Findings
Allen & Green (2012)	Consultants (*n* = 57) and Head Start staff (*n* = 407)	Cross‐sectional design HLM	Child: measures adapted from Green et al. (2006) (staff) Teachers: Head Start Mental Health Services Survey (staff, consultant)	Consultant report of positive relationship with teacher predicted staff report of positive relationship with consultant. The link between positive consultant relationships with families and positive Head Start staff relationships with consultants was stronger in urban than rural programs. None of the 5 consultant‐reported attributes predicted staff reports that IECMHC helped reduce children's behavior challenges. Consultant supervision, support, and knowledge of mental health best practices positively associated with staff‐reported child prosocial behaviors (at the level of a trend)
Beardslee et al. (2010)	Teachers (*n* = 60) and parents (324), classroom (*n* = 16)	Long‐term follow‐up (4 years, annual surveys and twice observations) Descriptive statistics	Teacher: CIS (trained observer), staff attendance (administrative records) Classroom: ECERS‐R (trained observers)	Increase in unused sick time during implementation period. Ratings of classroom quality and caregiver behavior towards children increased throughout the intervention, but there was no true baseline and ratings were already above national averages at the first time‐point
Bender et al. (2017)	Children in treatment (*n* = 247) and comparison group (*n* = 72)	Pre/post with comparison group; Baseline: during intake; Follow‐up: after completion of program (or equivalent 6‐month for control group) Multiple regression, mediation	Child: DECA (parents) Parents: PSI‐SF (parents)	CCEP associated with reduced parent‐child dysfunctional interactions and parent stress Parent‐child dysfunction (but not parent distress) mediated the relationship between IECMHC and child behavior problems
Conners‐Burrow et al. (2012)	Teachers (*n* = 193) and children (*n* = 1448)	Pre/post design; Baseline: beginning of school year; Follow‐up: end of school year ANCOVA	Child: DECA (teachers) Teacher: CIS (trained observer)	Teachers in the project reported high levels of satisfaction with consultation services and good relationships with consultants. Treatment differences were seen for 2 of 4 indicators of teacher behavior: less permissive (significant) and more positive (trend‐level). Years 1 and 2 yielded no significant group differences in child outcome posttest scores, but in Year 3, there was a significant difference between treatment and control sites on children attachment scores and behavior problems
Conners‐Burrow et al. (2013) [part of Conners‐Burrow et al. (2012) study]	Teachers (*n* = 115) from childcare centers (*n* = 18)	Pre/post design; Baseline: late August/September; Follow‐up: April/May Multiple regression	Child: DECA (teachers, parents) Teacher: CIS (trained observer)	Consultant time in the classroom was associated with less teacher punitiveness, permissiveness, and detachment, and greater use of positive classroom‐management skills (controlling for baseline behaviors). Frequency of consultant meetings with teachers was not associated with teacher‐child interactions, but was related to a reduction in teachers’ intent to leave the profession
Virmani et al. (2013) [part of Conners‐Burrow et al. (2012) study]	Teachers (*n* = 141)	Longitudinal design (average change score); Every 6 months (observation); up to 7 time points throughout 3 years (teacher reports) SEM path model (average change scores)	Teacher: CIS (trained observer)	This study demonstrated positive teacher‐child interaction gains in interaction, detachment, and punitiveness. The greatest improvements on average were seen for Detachment. Teacher assistants showed greater gains in quality than did lead teachers, but years of training, perceived training needs, and teacher preparation were not significantly associated with changes in interactions per year of exposure to IECMHC. The frequency of IECMHC activities was marginally and positively associated with less Detachment. Delivery of IECMHC (more frequent and constructive activities) and teacher experiences (positive relationship and perceived professional benefit) significantly predicted changes in quality of teacher‐child interactions
Conners‐Edge et al. (2020)	Children (*n* = 304)	Pre/post design; Baseline: before consultation; Follow‐up: after 3 months of consultation	Child: SDQ (teachers)	Teachers reported statistically significant decreases in behavior difficulties of the child who was the focus of consultation (e.g., conduct problems and hyperactivity), with small to moderate effect sizes. Teachers also reported significant improvements in the child's prosocial skills
Crusto et al. (2013)	Children (*n* = 261) and teachers (*n* = 14)	Pre/post design; Baseline: within first 4 months; Follow‐up: at program completion or shortly thereafter (6‐9 months after program initiation) Repeated ANCOVA	Child: DECA (teachers) Teacher: Self‐Efficacy Inventory (teachers)	All children in the CCP improved significantly on initiative (as measured by the DECA) and total protective factors, but there were no statistically significant improvements on children's self‐control or behavioral concerns. Children with low protective factors and/or high behavioral concerns showed the greatest improvements. Teachers’ self‐efficacy significantly increased
Davis et al. (2020)	289 teachers with 316 children and 62 consultants. Multilevel modeling	Pre/post design; Baseline: before beginning program; Follow‐up: 6 months into program Multilevel modeling	Child: STRS‐SF (teachers), DECA (teachers) Teacher: CCW‐JSI (teachers), TOS (teachers)	Higher quality relationships between consultants and teachers, or consultative alliance (CA), predicted greater growth in teacher, classroom, and child outcomes. Specifically, CA was predictive of positive teacher‐child relationships, children's attachment behaviors, and more positive classroom climate. CA was associated with teachers’ higher self‐efficacy and more positive job attitudes, such as job satisfaction
Gilliam et al. (2016)	88 classrooms 176 target children	RCT design; Baseline: within 2 weeks before intervention; Follow‐up: within 2 weeks after intervention HLM	Child: SSRS (teachers), Conners Teacher Rating Scale (teachers), PERM (teachers) Classroom: CLASS (trained observer)	ECCP treatment effects were only found for target children's externalizing scales after controlling for gender and pretest scores: teacher‐rated “acting out” behaviors were significantly lower in the intervention group. There were no significant differences between treatment and control for any domain of the CLASS. The ECCP treatment also had no impact on PERM factors
Green et al. (2012)	Staff (*n* = 124)	Pre/post design; Baseline: pre‐service training; Follow‐up: end of Year 2 HLM	Teacher: TOS (teachers)	Significant improvements in knowledge of best practices of early childhood mental health, decreased levels of job‐related stress, and feeling more supported by their program
Heller et al. (2011)	Consultants (*n* = 12), Teachers (*n* = 700)	Pre/post serial cohort design; Baseline: 4 weeks pre‐intervention; Follow‐up: within 4 weeks post‐intervention Random effects models	Teacher: TOS (teachers), GAS (teachers)	Teacher‐rated relationships with mental health consultants were high, with an average rating of 5.5 out of 6. The study also demonstrated an increase in scores between retrospective pretests and post‐tests for teacher self‐efficacy. The intervention yielded greater changes for younger, less experienced teachers compared to older, more experienced teachers
Heller et al. (2012) [part of Heller et al. (2012) study]	Teachers (*n* = 445) from childcare centers (*n* = 158)	Pre/post serial cohort design; Baseline: 4 weeks pre‐intervention; Follow‐up: within 6 weeks post‐intervention Mixed‐effects models	Classroom: CLASS (trained observer)	MHCs improved the quality of teachers’ interactions (e.g., emotional support and classroom organization) with children in their care. There were improvements between baseline and follow‐up on all 7 dimensions of the CLASS
Silver & Zinsser, 2020	Administrators (*n* = 124)	Cross‐sectional design with purposive sampling Logistic and linear regression	Teachers: CES‐D (teachers)	Findings indicate that teachers with greater levels of depression are more likely to request that a child be expelled from their care but that this association is attenuated by their centers’ utilization of infant/early child‐ hood mental health consultation services. IECMHC was not associated with reduced teacher depression scores, though it moderated the relationship between IECMHC use and expulsion requests
Upshur et al. (2009)	Intervention children (*n* = 47) and control children (*n* = 89)	Non‐equivalent control group design; Baseline: late fall (Oct/Nov); Follow‐up: late spring (May/June) Repeated ANOVA	Child: ESP (teachers), DP‐II (MHCs) Teacher: MBI (teachers) Parent: PSI‐SF (parents), PS (parents)	Intervention children improved significantly more than children at‐risk than children whose families refused or dropped out of services or who were true wait‐list controls. Improvement in children's behavior was specifically related to the number of intervention hours a child received. Children's developmental skills significantly improved post‐intervention and this improvement was independently associated with improvement in behavior. Overall rates of children being expelled and suspended from the 5 sites also decreased dramatically. The study found no significant effects on parent and teacher skills or well‐being, perhaps speaking to the trade‐offs between child‐focused and adult‐focused intervention. Teachers were more positive about services the longer their center had been receiving the intervention
Vuyk et al. (2016)	29 home‐based and center‐based child care providers in rural Kansas.	Longitudinal design; Collected at every session Multilevel growth modeling	Child: ultra‐brief measure of problem‐solving, prosocial behavior (providers) Teachers: Session Rating Scale 3.0 (teachers)	Child care providers shifted toward more professional growth over the course of the intervention with significant quadratic effects for time for personal well‐being, implementing activities and routines, connecting with parents, and more positive discipline. Providers’ perceptions of child behavior and well‐being outcomes also improved significantly over time and had significant quadratic effects for time. All models found that providers who improved the most were the ones who started out lower, as scores tended to even out due to a ceiling effect of the measures

Abbreviations: BPI, Behavior Problems Index; CCW‐JSI, Child Care Worker‐Job Stress Inventory; CES‐D, Center for Epidemiologic Studies‐Depression Scale; CIS, Arnett Caregiver Interaction Scale; CLASS, Classroom Assessment Scoring System; DECA, Devereux Early Childhood Assessment; DP‐II, Developmental Profile II; ECERS‐R, Early Childhood Environment Rating System‐Revised; ESP, Early Screening Project; GAS, Goal Attainment Scale; HLM, Hierarchical Linear Modeling; MBI, Maslach Burnout Inventory; PERM, Preschool Expulsion Risk Measure; PIPPS, Penn Interactive Peer Play Scale; PKBS‐2, Preschool and Kindergarten Behavioral Scales; PMHCS, Preschool Mental Health Climate Scale; PRSA, Preschool Self‐Regulation Assessment; PS, The Parenting Scale; PSI‐SF, Parent Stress Index‐Short Form; RCT, Randomized Control Trial; SSRS, Social Skills Rating System; STRS‐SF, Student‐Teacher Relationship Scale‐Short Form; TOS, Teacher Opinion Survey.

For this review, we define IECMCH‐related outcome measures as quantitative tools used to assess the impact of IECMHC at any ecological level. Studies may have used a variety of measures to capture information about potential mediator or moderating factors (e.g., satisfaction with service) but these are not included in Table 2 because they were not considered outcomes of the intervention. Similarly, qualitative outcome data are not included in Table 2. Notably, outcome measures are categorized by level of measurement (e.g., teacher) but many measures address various levels of the complex ecological system that is an ECCE program. For example, the Arnett Caregiver Interaction Scale (CIS; Arnett, 1989) could arguably be conceptualized as a teacher‐level, teacher‐child level, or classroom‐level measure. Because the informant is the teacher, we have categorized it at that level in Table 2. Coders were also instructed to code for the ecological level at which the study's outcomes were assessed (child, parent, teacher, classroom, school/center). A visual depiction of these findings can be found in Table 3.

**TABLE 3:** Synthesis of evidence across ecological levels

	**Bioecological levels**					
**Study**	**Child**	**Parent**	**Teacher**	**Classroom**	**School/center**	**Study design**
Allen and Green (2012)	∙ +			∙*	+	Cross‐Sectional
Beardslee et al. (2010)	+	∙ +	∙ +	+	∙*	Longitudinal, Mixed‐Methods
Bender et al. (2017)	∙*	∙*			+	Q‐E (Pre/post & comparison)
Conners‐Burrow et al. (2012)	∙*	+	∙* +			Q‐E (Pre/post & comparison)
Conners‐Burrow et al. (2013)	∙*	+	∙* +			Q‐E (Pre/post)
Virmani et al. (2013)		+	∙* +			Q‐E (Pre/post)
Conners‐Edge et al. (2020)	∙*					Q‐E (Pre/post)
Crusto et al. (2013)	∙* +		∙*	+		Q‐E (Pre/post)
Davis et al. (2020)	∙ +		∙ +	∙		Q‐E (Pre/post)
Gilliam et al. (2016)	∙* +	+	+	∙ +		RCT
Green et al. (2012)			∙* +		+	Q‐E (Pre/post)
Heller et al. (2011)	+	+	∙* +	+		Q‐E (Pre/post)
Heller et al. (2012)	+	+	+	∙* +		Q‐E (Pre/post)
Silver and Zinsser (2020)			∙ +			Cross‐sectional
Upshur et al. (2009)	∙* +	∙ +	∙ +	+	+	Q‐E (Pre/post & comparison)
Vuyk et al. (2016)	∙* +		∙*		+	Longitudinal, Mixed‐Methods

*Note*: ∙ indicates the level was evaluated; ∙* indicates at least one significant result; + indicates level was a focus of intervention.

Abbreviations: Q‐E: Quasi‐Experimental, RCT: Randomized Controlled Trial.

A secondary round of coding occurred to answer our final research question pertaining to the IDEAS Impact Framework. Two new independent coders within the authorship team were assigned to each of the 16 included articles. Coders were asked to indicate the extent to which each study attempted to address any of the guiding questions of the IDEAS Impact Framework: *What* about it works?; *How* does it work?; *For whom* does it work, and for whom does it *not* work?; *In what contexts* does it work? Specifically, coders reviewed each studies’ analyses to determine if they tested whether specific parts of the intervention, such as fidelity or dosage, were associated with differential outcomes (the “what”); whether mediating factors, such as changes in teacher attitudes, were examined (the “how”); whether child/teacher/consultant moderation outcomes were examined (the “whom”); and whether outcomes we examined across different contexts (the “where”).

Prior to this coding process, all five coders met virtually to practice coding one of the articles and answer any questions or challenges that might arise in interpreting the remaining articles. The coding team re‐convened after all articles were coded to discuss any discrepancies between coders or outstanding questions of individual coders. It should be noted that although the team coded 16 articles, they draw on data from only 13 unique studies (Table 2). In the following sections, papers included in this review are referred to using the first author and publication year. Full citations are marked with an asterisk in the references section.

## 3 RESULTS

The results below are organized to address the following study aims: 1) describe the characteristics of IECMHC interventions with published evaluations between 2009 and 2020 and the contexts in which they are being implemented; 2) describe the methods and measures employed in the evaluation of IECMHC interventions; 3) synthesize the evidence regarding the main effects of IECMHC across ecological levels; and 4) investigate the extent to which these modern evaluations align with the IDEAS Impact Framework.

### 3.1 Characteristics of evaluated IECMHC interventions

Findings related to our first research aim of describing the characteristics of reviewed interventions are summarized in Table 1. The interventions described in this paper generally adhere to characteristics outlined by Cohen & Kaufman (2005), for example, many IECMHC interventions included in this review were based on models that allowed for activity variation to best meet the needs of individual ECCE programs, and commonly employed consultant activities including observations at both the classroom and specific‐child level and universal classroom management skills training, often through the use of consultant modeling. Additionally, across most interventions, a central focus of IECMHC was adults, both teachers and parents. Five interventions provided direct support to parents, through education and services. In support of teachers, staff training was part of four interventions and one intervention provided specific supports around staff wellness and stress management.

#### 3.1.1 Duration and dosage

As can be seen in the right‐most column of Table 1, duration and dosage varied widely within this sample, and their combined variation in total duration, length of individual visits, and frequency of visits. For example, while Gilliam et al. (2016) described the shortest duration intervention (2 months), participating preschool programs received on average 4–6 hours of direct service each week during that period making it the highest dosage intervention in the analysis set. Looking across the sample, eight interventions had durations under one year and dosages of between one (Bender et al., 2017) and 6 h per week (Heller et al., 2011, 2012). Three other studies described interventions that stretched over three years. Beardslee et al.’s (2010) program included consultation two times per week for 3 years and Conners‐Burrow et al. (2012) and Virmani et al. (2013) utilized a half‐day per week model for 3 years. It should be noted that the variability of reporting regarding duration and dosage makes it impossible to consistently compare service hours across studies.

There was also great variability in timing and dosage between ECCE programs. In one intervention, the number of sessions ranged from 1 to 16 and the number of weeks between the first and last sessions ranged from 2 to 19 weeks (Vuyk et al., 2016). In another intervention, teacher turnover and new hires led to variation in a teacher's length of time in the intervention and the number of data time points (Conners‐Burrow et al., 2013; Virmani et al., 2013).

### 3.2 Methods used in IECMHC evaluation studies

Findings related to our second research aim of describing the methodologies employed in evaluating IECMCH interventions are summarized in Table 2.

#### 3.2.1 Study designs

Of the 16 peer‐reviewed studies included in this review, only one used a randomized control trial design (Gilliam et al., 2016). An additional three studies employed quasi‐experimental methods with pre/post non‐equivalent control groups. Upshur et al. (2009) compared children referred for services who were non‐randomly assigned to either receive the intervention or to be in the comparison group. Bender et al. (2017) recruited families from counties that did not offer IECMHC to serve as a control. In the study by Conners‐Burrow et al. (2012) intervention sites were matched to sites with similar demographics that did not receive consultation services (Conners‐Burrow et al., 2012). Aside from one study which used a cross‐sectional, single‐time point design (Allen & Green, 2012), the remaining studies used pre‐post or longitudinal designs with an IECMHC cohort. Two studies, based on a single intervention, utilized a pre/post serial cohort design to account for threats to validity such as maturation and external events, with one of the studies using an additional retrospective pretest to address response shift bias (Heller et al., 2011, 2012). Only one study used a longitudinal design with data collection occurring at up to seven unique time points (Virmani et al., 2013).

#### 3.2.2 Approaches to measurement

Studies in this review used a variety of measures, both standardized and unpublished, to assess the impact of IECMHC at various levels. The name of the measures, informants, and ecological levels are summarized in Table 2. Of the eight studies that included child outcomes, the majority used teacher‐report only, however, two captured child behavior from multiple informants (e.g., teachers and consultants, or parents and trained observers).

Trained observers were used to measure teacher‐child relationship quality via the Arnett Caregiver Interaction Scale (CIS; Arnett, 1989) in four studies. Trained observers were also used to assess classroom quality in three studies using either the Classroom Assessment Scoring System (two studies; CLASS; Pianta et al., 2008) or the Early Childhood Environment Rating Scale‐Revised Edition (one study, ECERS‐R; Harms et al., 1998).

Self‐report measures were also used to capture teacher‐level outcomes in six studies, and parenting outcomes in two studies. Lastly, one study uses administrative staff attendance records to evaluate staff absenteeism as an outcome (Beardslee et al., 2010). None of the studies reviewed gathered quantitative administrator‐, programmatic‐, or center‐level outcome measures.

### 3.3 Synthesis of evidence across ecological levels

Table 3 summarizes findings related to our third aim of synthesizing the evidence of the effects and associations of IECMHC on children, adults, and programs.

#### 3.3.1 Child‐level outcomes

Of the 16 peer‐reviewed articles, half (*n* = 8) present varying degrees of compelling evidence for the positive role of IECMHC on children's behavioral and social outcomes, with several studies also linking IECMHC to declines in expulsion risk. A total of eight studies demonstrate at least some reduction in children's internalizing and externalizing behaviors or an increase in children's social skills (Bender et al., 2017; Conners‐Burrow et al., 2013, 2012; Conners‐Edge et al., 2020; Crusto et al., 2013; Gilliam et al., 2016; Upshur et al., 2009; Vuyk et al., 2016). Further, Davis and colleagues (2020) identified a relationship between consultative alliance and children's attachment behaviors.

After controlling for gender and pretest scores, Gilliam et al. (2016) found only treatment effects for target children's externalizing behaviors. Conners‐Burrow et al. (2012) found improvements in children's behavior, as measured by the Devereux Early Childhood Assessment (DECA), in year three of their intervention. Another study found that the intervention improved the initiative and total protective factors of the children evaluated, also measured by the DECA, but found no statistically significant improvements in children's self‐control or behavioral concerns (Crusto et al., 2013). Upshur and colleagues (2009) also found significant improvements post‐intervention in developmental skills for the children who received the intervention.

Three studies report findings regarding expulsion or expulsion risk. One study found an association between IECMHC and expulsion rates based on administrative data (Upshur et al., 2009), and another study found an interaction between IECMHC and teacher depression such that teacher depression was no longer positively associated with expulsion requests when they were receiving IECMHC (Silver & Zinsser, 2020). In terms of expulsion risk, Gilliam et al. (2016) found no treatment impact on any of the subscales of the teacher‐report Preschool Expulsion Risk Measure (PERM): classroom disruption, fear of accountability, hopelessness, and teacher stress.

#### 3.3.2 Parent‐level outcomes

Two studies assessed parent‐level outcomes using the Parenting Stress Index‐Short Form; one of which found IECMHC to be associated with reductions in parenting stress (Bender et al., 2017) and the other found no improvements on parent stress or well‐being (Upshur et al., 2009). It should be noted that Conners‐Edge et al. (2020) included a measure of parental engagement in the IECMHC process, but does not report on its association with outcomes.

#### 3.3.3 Teacher‐level outcomes

Seven studies reported at least one significant positive teacher‐level outcome, including job stress, competence and knowledge, depressive symptoms, teacher efficacy, and teacher‐child interactions. IECMHC was associated with improvements in teacher job stress (Green et al., 2012; Vuyk et al., 2016) and competence and knowledge (Beardslee et al., 2010; Green et al., 2012). Although Silver & Zinsser (2020) did not find IECMHC to be related to depressive symptoms for teachers, teacher depression did moderate the relationship between IECMHC and their requests for expulsion. Three studies also reported improvements in teachers’ self‐efficacy with respect to addressing children's social‐emotional and behavioral needs as a result of consultation (Crusto et al., 2013; Davis et al., 2020; Heller et al., 2011). Of the four studies that measured teacher‐child interactions, two reported positive outcomes. Conners‐Burrow et al. (2012) reported that treatment teachers were significantly less permissive and more positive (trend‐level significance) than control teachers. Teachers in Virmani et al. (2013) were less punitive and detached following consultation. The majority of studies assessing teacher well‐being found significant effects in the expected direction. One study (Upshur et al., 2009) reported insignificant results, but this was likely due to the child‐level emphasis of their particular intervention model.

#### 3.3.4 Classroom‐level outcomes

Of the three studies assessing aspects of the classroom environment, two reported significant positive outcomes. Heller et al. (2012) reported significant improvements across all three domains of classroom quality as measured by the CLASS over the study period. Conversely, the treatment group receiving IECMHC and the control group in Gilliam and colleagues’ study (Gilliam and Shahar, 2006) did not significantly differ on any CLASS domain. Another study, using the ECERS‐R and teacher reports, found improvements in classroom quality (Beardslee et al., 2010).

### 3.4 Alignment between IECMHC evaluation studies and IDEAS impact framework: Identifying what, how, whom, and where?

The final aim of this study was to review the current literature base in light of modern conceptualizations of program evaluation using the IDEAS Impact Framework (Center for the Developing Child et al., 2016). No study in this review fully addressed all of the framework's guiding questions and seven studies addressed none. Below we summarize the findings of this review by each guiding question.

#### 3.4.1 What: Identifying characteristics of the intervention associated with differential outcomes

Three studies tested whether intervention dosage (time in the classroom and frequency of meetings with teachers) was associated with teacher‐ or child‐level outcomes. Conners‐Burrow et al. (2013) found that when consultants spent more time in the classroom, teachers were less likely to consider leaving the early childhood profession. Frequency of consultation activities was also associated with better teacher‐child interactions (lower detachment; Conners‐Burrow et al., 2012; Virmani et al., 2013). Only one study tested the association between dosage and child outcomes. Upshur et al. (2009) found that the children's behavior change was positively associated with the number of hours of consultation. A few articles described the fidelity of implementation of the IECMHC model and consultation activities (e.g., Beardslee et al., 2010; Heller et al., 2011); however, no results examined the association between implementation fidelity and teacher‐level or child‐level outcomes.

#### 3.4.2 How: Identifying what factors may mediate the relationship between IECMHC intervention and outcomes

Several potential mediating factors were identified in the reviewed studies, including factors across ecological contexts (e.g., consultant, teacher, parent‐child). Other potentially mediating factors examined related to teachers were the teacher's quality of experience with IECMHC, which predicted changes in the quality of teacher‐child interactions (Virmani et al., 2013), and the strength of the consultative alliance between consultants and teachers, which predicted improvements in teacher‐child relationships, children's attachment behaviors, and classroom climate first 6 months of consultation (Davis et al., 2020). Of note, Upshur et al. (2009) found that changes in child development skills did not mediate the relationship between IECMHC service hours and child behavior change. Finally, one study examined potentially mediating parent factors, finding that the presence of parent‐child dysfunction (but not stress) mediated the positive association between IECMHC and child behavior problems, as well as protective factors (Bender et al., 2017). It should be noted, however, that although many of the evaluations examined potentially mediating factors, only two (Bender et al., 2017; Upshur et al., 2009) examined a full mediation model linking IECMHC to child outcomes through the mediators.

#### 3.4.3 Whom: What characteristics of children, teachers, and consultants moderate the relationship between IECMHC and outcomes

A handful of the studies included in this review examined whether there was a differential link between IECMHC and outcomes based on child, teacher, and/or consultant characteristics. Specifically, we identified mixed results across this set of studies identifying teacher, child, and family characteristics that were associated with greater program impacts.

Several studies found that teacher‐level characteristics were related to differential findings of IECMHC. For example, Silver & Zinsser (2020) identified that teacher depression moderated the relationship between IECMHC use and expulsion requests, such that the association between teacher depression and expulsion requests was only significant in the absence of IECMHC. Teacher‐reported changes in professional growth observed instructional quality was greatest for teachers who had lower baseline scores (Virmani et al., 2013; Vuyk et al., 2016). Likewise, Heller et al. (2011) reported that the greatest improvements in teacher self‐efficacy were seen for teachers who were younger and less experienced and Virmani et al. (2013) identified that teacher assistants had greater gains in quality of teacher‐child interactions compared to lead teachers, although years of experience was not associated with quality in this study.

Similar to the teacher factors discussed above, child‐level behaviors were seen as predictive of the strength of the intervention effects where children who scored with higher behavioral concerns at baseline had the greatest improvements from IECMHC (Crusto et al., 2013; Upshur et al., 2009). Crusto et al. (2013) found that children with low protective factors and/or high behavioral concerns had the greatest improvements, and similarly, Upshur et al. (2009) identified that boys with poorer baseline behavior scores and developmental skills had more improvements in follow‐up behavior scores. Of note, the Upshur et al. (2009) article was the only one to identify child demographic characteristics (e.g., child sex) as a potential moderator.

One study included in this review (Allen & Green, 2012) specifically examined the attributes of the consultants themselves. Although they did not examine whether these factors specifically moderated the relationship between IECMHC and child/teacher outcomes, they did find a relationship between consultant attributes and potential mediators of the relationship between IECMHC and outcomes. More specifically Allen & Green (2012) identified some marginally significant associations between the vast majority of consultant attributes (including knowledge of child development and experience with Head Start, relationships with families, relationships with staff, training, supervision and support, and knowledge of ECMH best practices) and staff reports of positive relationships with the consultant, although these consultant attributes were not related to staff reports that consultation helped reduce behavior challenges.

### 3.5 Where: In what contexts does IECMHC work?

Although the studies reviewed represented a range of different settings, only two studies examined whether there was moderation at the program/community‐level, in other words examining whether there were differential effects of IECMHC across different contexts. Allen & Green (2012) examined whether program location (rural/urban) moderated the association between the consultant's relationship with a parent and the direct service staff's relationship with the consultant, where this relationship was stronger for urban programs compared to those in rural settings. Examining a different aspect of context, Vuyk and colleagues (2016) found that home‐based providers showed more satisfaction with IECMHC compared to center‐based providers, all located within a rural setting.

## 4 DISCUSSION

The purpose of this systematic review was to provide an updated synthesis of the evidence base for Infant and Early Childhood Mental Health Consultation (IECMHC) in the past ten years, since the publishing of two prior reviews by Brennan et al. (2008) and Perry et al. (2010). The substantial number of studies published since prior reviews provide justification for the need to systematically gather and synthesize the expanding evidence. Our review yielded three main findings: modern IECMHC studies evaluated a wide array of IECMHC interventions representing different levels of intervention delivery hours; findings from these studies continue to build evidence of the efficacy of the intervention at multiple levels, but teacher‐ and child‐level measures still predominate; and finally, despite their recent publication, these studies are only just the beginning of aligning IECMHC evidence with modern conceptualizations of program evaluation and cannot yet provide a full or nuanced explanation of the active ingredients or contextual influences on IECMHC impact. In this discussion, we will further describe these results and place them in the context of suggested next steps for the field.

### 4.1 Diverse approaches, common outcomes

Our first research aim pertained to describing the characteristics of IECMHC interventions with published evaluations between 2009 and 2020. This systematic review revealed that IECMHC is implemented in states and localities across the United States, and there is considerable variability across interventions in terms of consultation length, target population, and activities employed, to name a few. The diversity of IECMHC structures was reflected in the 16 articles identified in this systematic review. Further, and related to our second research aim, the included studies differed in their methodological approaches, utilizing a range of measurement tools, research designs, and data collection timelines. This diversity of approach differs from many evidence‐based practices which are more easily evaluated because they have centralized leadership that distributes training, interventional materials, and fidelity measures. It is striking, therefore, that there is considerable consistency in findings across studies, as well as consistency with findings from the two prior reviews. Our third research aim sought to summarize the main effects of IECMHC interventions at each level of the ecological model, starting with findings related to children's outcomes.

Children, the indirect beneficiaries of consultation, typically are referred for consultation because they demonstrate externalizing behaviors, which has been associated with increased risks of being expelled (Perry et al., 2011). All of the studies (*n* = 4) that measured child externalizing behavior demonstrated statistically significant reductions in these behaviors. Specifically, IECMHC was shown to predict reductions in aggression, hyperactivity, and problem behaviors, among others. Because ECCE teachers are often not sufficiently trained to manage challenging behaviors (Snell et al., 2012), which are the most common explanation for ECCE expulsions (Perry et al., 2011), this finding is of key clinical and policy significance. It is important to note, however, that none of these studies measured internalizing behaviors. While they are less likely to lead to expulsion, internalizing behaviors in young children may indicate exposure to stress or trauma and may also warrant mental health consultation. While internalizing behaviors were shown to decrease after IECMHC in Raver et al.’s (2009) study, which was included in Perry et al.’s (2010) review, this remains an understudied outcome.

In a similar vein, in all of the studies in which child social‐emotional competencies were measured, children demonstrated at least one significant improvement. Significant growth was seen across a range of constructs: initiative, attachment, prosocial behavior (Vuyk et al., 2016), and adaptive behavior (Upshur et al., 2009), though several null findings for self‐control were reported. These results are consistent with the strengths‐based approach of IECMHC. Child social‐emotional competencies are key outcomes because they are critical for school readiness and may buffer children against stressors and future mental health issues (Bagdi & Vacca, 2005). Importantly, most studies relied upon teacher‐reported changes in both challenging behavior and prosocial behavior. This is a key limitation because successful interventions generalize across multiple contexts (e.g., school and home) and parent and teacher reports have been shown to differ significantly (Dirks et al., 2012).

Further, reduction in ECCE expulsions is a key policy lever for advocating for IECMHC funding, so it is particularly important to evaluate the evidence for IECMHC impacting this disciplinary practice. Three of the studies addressed this outcome, but each in a markedly different manner; no study in this review demonstrated that individual children who engaged in IECMHC were less likely to be expelled. Rather, studies reported overall reductions in rates of expulsions for centers that received consultation (Upshur et al., 2009), as well as reductions in the frequency of teachers requesting that a child be expelled (Silver & Zinsser, 2020), but no impact on children's risk for expulsion (Gilliam et al., 2016).

It is important to note that prior studies have supported the claim that individual children engaged in IECMHC are less likely to be expelled (Davis et al., 2020; Perry et al., 2008), and that their expulsion risk is attenuated (Shivers et al., 2019). Overall, these findings should be interpreted in light of the difficulty of measuring expulsion. Scholars have noted the limitations of various approaches to measurement, including “soft expulsions,” differences in parent and ECCE staff responses, and reluctance for centers to acknowledge expulsions, particularly when prohibited by local policies (Meek & Gilliam, 2016). This methodological challenge speaks to the need for systematic and mandatory statewide data systems across ECCE. Consistent with Albritton et al. (2019), this review did not identify any empirical support that disparities in expulsion rates are reduced after consultation. Young children of color are at increased risk for expulsion (Gilliam, 2005), yet research has not yet demonstrated whether IECMHC may close this gap.

Of note, while there was general consistency in child‐level outcomes across many of the studies, this was only partially reflected in the consistency of intervention focus. As is seen in Table 3, the various IECMHC interventions focused on different targets, some focusing exclusively on child targets, some on teachers, and some on multiple targets of intervention. In some cases, as might be expected, the target of the intervention was aligned with the measured and identified outcome. For example, 9 of the 11 studies that measured teacher outcomes also had a teacher target built into the intervention. However, of the nine interventions that had a parent focus, only two also measured a parent outcome. The same inconsistency was seen for child outcomes in which nine interventions had a child focus and 10 studies measured child outcomes, but there was only overlap (e.g., child‐focused intervention and child‐level outcomes) among six studies.

### 4.2 Outcomes beyond the child

In line with our third aim, we also examined the impacts of reviewed studies related to higher levels of the ecological model. While children are the indirect target for IECMHC, teachers and staff directly work with the consultant and demonstrate a range of outcomes that are thought to lead to child outcomes. The studies in this review consistently measured teacher outcomes and clear findings emerged that IECMHC is associated with increased teacher knowledge, improved self‐efficacy, and more positive interactions with children. In other words, teachers grow in their knowledge and confidence to interact with children in ways that promote mental health and foster development. These findings are critical for several reasons. First, positive, nurturing relationships with ECCE teachers are thought to be key buffers for young children who experience toxic stress and are thought to be avenues for increasing equity in education (Shonkoff, 2012). In addition, teachers who feel stressed and powerless in response to challenging child behaviors are more likely to resort to expulsion (Gilliam & Reyes, 2018; Martin et al., 2018). The IECMHC findings to date regarding exclusionary discipline also further imply that the intervention is working at the systemic, center‐wide level, not just for an individual child displaying challenging behaviors.

It is important to note that teacher‐level findings are somewhat less consistent than child‐level findings and seem to vary based on the measures, sample, and research design. Importantly, Gilliam et al.’s (2016) study reported null teacher and classroom effects. This finding is puzzling because, as an indirect intervention, changes in teacher knowledge, attitudes, and behaviors are thought to precede changes in children. As the IECMHC intervention with the shortest duration (4–6 weeks) in this review, it is possible that teachers did not demonstrate and report measurable changes in that short period or that they were experiencing an “implementation dip” (Fullan, 2001), while at the child level, a more sensitive measure may have been used. Nevertheless, in studies of consultation programs of varying durations, there were occasional null effects on teacher outcomes, including behavior management skills and punitive interaction styles (Conners‐Burrow et al., 2012; Upsher et al., 2009).

While several studies included in this review documented program‐level changes as a result of IECMHC, most did not measure changes at the program level. This is a major limitation, perhaps reflecting both lack of sufficient attention to systemic issues or lack of adequate measurement tools to assess change in program practices, policies, and climate.

### 4.3 IDEAS Impact Framework alignment

In many ways, the child and teacher outcomes described thus far echo findings from the two prior reviews of IECMHC (Brennan et al., 2008; Perry et al., 2010). It is affirming that the positive impacts are found consistently over time using some novel research methods and different measurement tools. A novel contribution of the present review is to expand our conceptualization of evidence of effectiveness. Specifically, each of the reviewed studies were additionally coded based on whether they attempted to address any of the four key questions outlined in the IDEAs Impact Framework (Center for the Developing Child et al., 2016). In general, there was relatively limited analytical attention to the mediation and moderation questions posed in the framework. No study addressed all four and many addressed none of the guiding questions. Several studies reported collecting data that would have allowed for these more nuanced analyses (e.g., Conners Edge et al., 2020; Vuyk et al., 2016), but they were not reported.

Given the attention to early childhood expulsion in the framing of several of the studies reviewed, it was also surprising to find that none reported on whether there was a differential impact of IECMHC on children based on indicators shown to be predictive of expulsion, such as race/ethnicity, gender, and disability. In fact, Crusto et al.’s study (2013) was the only one in this review to analyze differential impact for children, reporting that children scoring low in protective factors and/or high behavioral concerns at baseline demonstrated the greatest improvements from IECMHC. When the “for whom” question was asked at the teacher‐level, IECMHC appeared to have a stronger impact when implemented with teachers with lower scores on measures of teaching practices (Vuyk et al., 2016) and those who are younger and less experienced (Heller et al., 2011). While none of the programs in this review stated that they were purposefully allocating resources to children and teachers with indicators of greater need, the growing literature on moderators of IECMHC suggests this would lead to the largest impact; further, it would align with the goal of IECMHC to address disparities.

Research attending to how IECMHC works was also lacking. Multiple studies affirmed that higher “doses” of consultation predicted stronger effects; however, those who received more consultation may also have had more severe problems at baseline, making determinations about standardized doses of consultation difficult. One study (Bender et al., 2017) in this review conducted mediation analyses, concluding that dysfunctional parent‐child interactions significantly mediated the relationship between IECMHC and child behavioral problems as well as child protective factors. While mediation analyses are scarce in the IECMHC literature, they have been conducted in other studies included in prior reviews (Green et al., 2006). Further, several studies in this review investigated constructs that are hypothesized mediators in IECMHC, though they did not demonstrate statistically that those constructs served a mediating role. For instance, Davis and colleagues (2020) demonstrated that a stronger consultative alliance (i.e., the consultant‐consultee relationship) predicted greater 6‐month improvements in outcomes such as teacher‐child closeness, child attachment behaviors, and classroom climate. Importantly, other studies that were not included in the current review have provided rich, qualitative data on the importance of relationships in IECMHC. Kniegge‐Tucker et al. (2020) analyzed teacher interview data to characterize common themes in the progression of the consultant‐consultee relationship and Denatale (2013) explored barriers and facilitators of a positive relationship between consultants and ECCE directors. While recent studies have begun to investigate the mechanisms of action for IECMHC, this area of study remains quite underdeveloped, both in terms of the constructs measured and the sophistication of analyses used.

This review focused solely on empirical evidence for IECMHC programs in early childhood care and education (ECCE). This focus reflects not only a desire for specificity but also the dearth of evidence for IECMHC in other contexts, despite the need for such evidence according to the IDEAS Impact Framework. IECMHC is a flexible intervention that has been integrated into a range of systems in which infants and young children are served, including home visiting, child welfare, primary care, and Early Intervention. At the time of this review, the only setting other than ECCE in which IECMHC had been evaluated was home visiting. In the one peer‐reviewed article reporting data on consultation in home visiting, Lambarth & Green (2019) conducted a mixed‐methods investigation of a pilot hybrid model in which home visitors were supported with both IECMHC and Early Childhood Positive Behavioral Interventions and Supports (ECPBIS). The 12 home visitors in the sample who worked with a consultant demonstrated improved knowledge of child and adult mental health (but not increased self‐efficacy in those domains) and improved confidence in partnering with parents, but did not demonstrate a significant reduction in stress. To better align with the IDEAS Impact Framework, a great deal more research into IECMHC across contexts is needed.

### 4.4 Limitations

Though this study is a necessary contribution to the field of IECMHC, it is not without limitations. First, several studies were excluded from this review because they implemented IECMHC in conjunction with other interventions or services and effects could not be isolated. The use of IECMHC as a supportive mechanism or framework for other ECCE enhancements is worthy of study in its own right but the findings of this review may not generalize to those combined approaches. Second, this review only focused on evidence published in peer‐reviewed academic journals and therefore does not encompass any “gray” reports published elsewhere. At the same time, many of these reports employed rigorous methods and are already being used to inform practice and policy progress nationwide. A third limitation is a reflection of the shortcomings of the designs of the studies reviewed. The majority of child outcomes reported across these studies utilize teacher reports and this is somewhat problematic given that one of the main targets of change in IECMHC is teacher perceptions of child behaviors. However, while we might not be able to decipher whether child behavior or teacher perceptions were being impacted by IECMHC, it is likely a combination of the two, and the work of changing teachers’ perceptions of children's behavior and helping them contextualize behavior in terms of their broader understanding of experiences, trauma, and implicit biases may actually be the most critical outcome of IECMHC.

Finally, there is considerable variability in the strength of the research designs of the studies included in this review. Although this is reflective of the overall challenges in rigorously evaluating the complex, multifaceted nature of IECMHC, it limits our ability to make broad generalizations of patterns across the study. Due to the small literature base, we have chosen to make note of general patterns regardless of the specific strength of the research design. As IECMHC research continues, ideally with an eye toward rigorous, randomized trials, we anticipate that future research can more fully take into account variations in research designs when considering variations across study outcomes.

### 4.5 Future directions and implications

While the evidence for IECMHC has begun to expand beyond quantifying outcomes to beginning to explore moderators and mediators, as outlined in the IDEAS Impact Framework, there are many additional research questions to be answered. Broadly, the study settings should over time more closely reflect the implementation settings; IECMHC takes place not only in ECCE, but also in a range of settings including home visiting, primary care, child welfare, and others. Given the unique approaches and priorities of these contexts, each must have its own evidence base to adequately answer the question “*where does it work?*”. Nevertheless, similar mechanisms of change are thought to apply across all settings. Additional measure development studies of the hypothesized driving forces of IECMHC (e.g., relationship between consultant and consultee, organizational change, etc.), as well as analyses (e.g., mediational models, path modeling), are needed to assess these mechanisms empirically. Further, to align with dissemination and implementation research, it is important to develop and use a measure of fidelity for IECMHC. Such fidelity measures will likely be unique to each individual iteration of IECMHC, but will be critical to understanding outcomes, or lack thereof. To align with policymakers’ priorities, it is important to assess the sustained impact of IECMHC with follow‐up data for consultees and to articulate the necessary and sufficient “dose” (i.e., duration and frequency) of consultation.

It is notable that none of these studies explicitly measure the impact of IECMHC on disproportionality in child outcomes or disaggregated findings by race/ethnicity, gender, or other variables. As such, a critical unanswered question from the IDEAS Impact Framework remains: *for whom does it work?* The next generation of research needs a more explicit equity focus whereby samples include higher percentages of children and programs from historically marginalized communities and demographic groups. The possible impact of IECMHC on closing disparities should be explored based on a wide range of child characteristics (e.g., race/ethnicity, gender, age, disability, child welfare involvement, linguistic background), as well as caregiver and program‐level variables.

As demonstrated in this systematic review, most key findings are consistent across time and program design. It has been established that IECMHC delivered in ECCE settings is associated with improvements in child challenging behavior and social‐emotional competencies, as well as teacher self‐efficacy, interaction styles, etc. The results presented here echo results from two prior, but dated, reviews of the evidence for IECMHC (Brennan et al., 2008; Perry et al., 2010). The current study lends considerable additional credibility given the sole reliance on peer‐reviewed studies; the relative consistency of findings across a range of outcome measures; and the use of increasingly sophisticated research methods. In addition to replication, the most recent decade of evidence begins to unpack *what works for whom* (IDEAS Impact Framework) in consultation. The next decade of research is likely to continue to clarify these questions, to speak to the evidence for IECMHC in varied settings, and to answer questions about the role of IECMHC in promoting equity.

## Data Availability

No.
